# SeCaT-meter 600: Prism-based dual channel optical density meter with self-calibration

**DOI:** 10.1016/j.ohx.2026.e00820

**Published:** 2026-08-01

**Authors:** Gregor Michl, Christoph Otto, Thomas Walther, Nandor Ziebart

**Affiliations:** Technische Universität Dresden, Institut für Bioverfahrenstechnik, Dresden, Germany

**Keywords:** Optical density, Photometer, Arduino

## Abstract

Accurate and real-time monitoring of optical density (OD) is one of the key requirements for control of microbial fermentations. It provides insight into current cell concentration and growth dynamics of microbial cell cultures by measuring optical density at 600 nm (OD_600_). In combination with additional chemical (e.g. pH, dissolved oxygen), and physical (e.g. temperature) parameters, adjustments of culture conditions can be made during cultivation. Optical density is determined through either in-line, on-line or off-line measurements, although on a laboratory scale manual sampling (off-line) is typically performed due to the high acquisition costs of on-line devices. UV/Vis spectrometers or photometers are generally used for this purpose, but these are not suitable in resource-limited settings or for small-scale laboratories or field applications due to their acquisition costs and the limited possibility of self-performed repairs and modifications. Using widely available electronic components and 3D printing, we designed a self-calibrating photometer capable of precise OD_600_ measurements for bioprocess monitoring. This device is intended to offer an affordable and robust solution for both laboratory-scale experiments and field applications, where portability and ease of use are paramount.

## Graphical abstract

**Figure:**
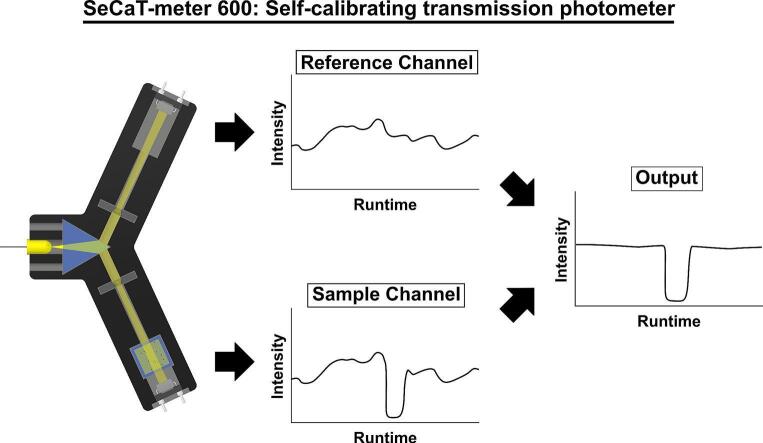

Specifications table (Table 1)

**Table 1:** Specifications table of the SeCaT-meter 600.

Hardware name	Self-calibrating open source OD_600_ photometer
Subject area	Chemistry and biochemistry Biological sciences (e.g., microbiology and biochemistry) Environmental Science Education
Hardware type	Measuring physical properties and in-lab sensors Field measurements and sensors
Closest commercial analog	Implen OD600 (911 €)
Open source license	CC-By Attribution 4.0 International
Cost of hardware	145 €
Source file repository	https://doi.org/10.17605/OSF.IO/E46QY

## 1 Hardware in context

Cultivation processes in bioprocess engineering typically span over several hours or days. On a laboratory scale, process monitoring is commonly performed via determination of the optical density at 600 nm (OD_600_). This value corresponds to the negative decadic logarithm of the transmitted light intensity *I* of a sample solution relative to a reference sample *I_0_* (Equation (1). At this wavelength most microbial cells and culture media exhibit insignificant absorption. Thus, reduction of transmitted light is primarily due to scattering effects, providing a rapid method to quantify cell concentration or biomass density. As such, OD_600_ measurement has become the reliable standard method for monitoring bacterial growth [1], [2].

(1) $${\mathrm{OD}}_{600}=\;-\log\frac{I_{600}}{I_{0,\;600}}$$

When measuring optical density by transmission, it is crucial to consider that linear correlation between cell density and OD_600_ applies only to low optical densities (OD_600_ < 0.4) [3]. At higher values, in-line and online optical density-measurements require side-scatter or backscatter techniques. In a laboratory-scale setting, high OD_600_ values are often determined by diluting sample solutions to low optical densities. These are typically investigated either with spectrometers or photometers, which quantify light intensity at one or multiple discrete wavelengths. Photometers represent a cost-effective alternative to spectrometers, attributed to their lower structural complexity. Unlike spectrometers, which include expensive optical components, such as a broad-spectrum light source, mirrors, lenses, and prism or grating monochromators, photometers often employ simpler and less costly components, such as light-emitting diodes (LEDs) and photodiodes [2], [4].

Increased accessibility and reduced cost of 3D printing as well as the widespread availability of inexpensive electronic components have led to multiple publications describing the construction of low-cost open-source spectro- or photometers [5], [6], [7]. These devices typically operate on a single-beam setup, utilizing LEDs as light source. In bioprocess engineering, optical density is used during the cultivation of microorganisms to determine cell density and growth phase, among other things. However, OD_600_ measurements in bioprocesses are typically performed over several hours or days, during which light-intensity drifts of LEDs require repeated recalibration to ensure accuracy of the measurement.

**Fig. 1:**
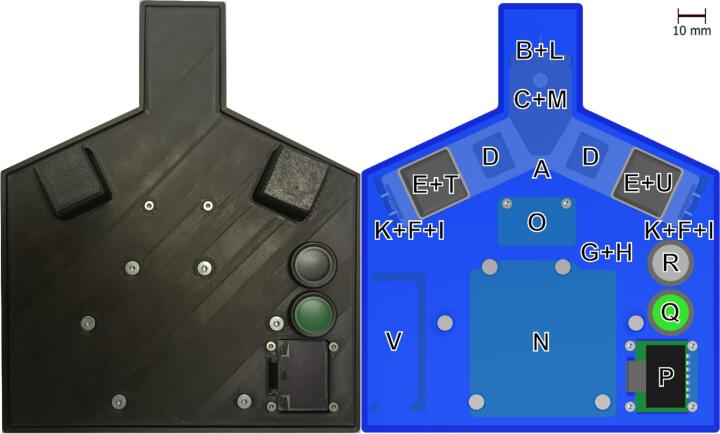
Top-view picture of the SeCaT-meter 600 (left), and rendered image with transparent housing, created with Autodesk Inventor 2026 with annotated parts (right). Main unit (**A**), LED holder (**B**), prism lid (**C**), pinhole apertures (**D**), cuvette caps (**E**), IC-socket holders (**F**), housing (**G**), housing lid (**H**), IC socket (**I**), OPT101 photodiode (**K**), LED600-03 (**L**), equilateral prism (**M**), Arduino Nano V3 + Nano Expansion shield (**N**), ADS1115 (**O**), SSD1306 OLED (**P**), measurement/bottom channel push-button (**Q**), reference/top channel push-button (**R**), measurement/bottom channel (**T**), reference/top channel (**U**) and 9 V block battery compartment (**V**).

In this study, we present the design and commissioning of a modular, low-cost, dual-channel transmission-photometer, equipped with a beam splitter for self-calibration of light intensity, named the SeCaT-meter 600 (Fig. 1). This configuration aims to enhance measurement reliability over extended cultivation periods, addressing key limitations of existing single-beam, low-cost systems.

With the presented device, we proved comparable performance of the SeCaT-meter 600 against a commercial spectrometer for the growth of *Saccharomyces cerevisiae* regarding accuracy, sensitivity, resolution, and measurement time.

## 2 Hardware description

### 2.1 General

The construction of a photometer consists traditionally of a light source, monochromator or optical filter, sample chamber, detector, and measurement unit with an integrated display. The core element of the DIY photometer, the SeCaT-meter 600 presented here, is the equilateral prism (**M**) positioned in between the LED light source (**L**) and the pinhole apertures (**D**). This setup allows for uniform beam splitting of the emitted LED-light into a reference- and a sample-beam, which are detected by two separate photodiodes.

This allows either for two separate samples being measured (*Independent Mode*) or real-time correction of environmental influences on measured voltages in one channel over extended operating periods without recalibration (*Self-Calibration Mode*). Thus, manual recalibration, either due to recording OD_600_ of two different bioprocesses or to accommodate for external influence is avoided, which is a common limitation in low-cost, single-beam systems. The criteria for the chosen electronic components were commercial availability, affordability and ease of use. The result is a robust and accessible design, which can be assembled by untrained personnel. It is suitable for long-term bioprocess monitoring applications, while maintaining a minimal technical and financial entry barrier for laboratory use and educational purposes. It is equipped with a 9 V block battery compartment (**V**) for mobile use. The total cost of all components is €145.

### 2.2 Main unit

Control of electronic components, acquisition and processing of signals in the dual-channel photometer are carried out using an Arduino Nano V3 microcontroller. The 3D-printed housing lid is designed to accommodate the expansion shield (**N**), which adheres to the standard Arduino footprint and can be easily replaced by other microcontroller systems. In this setup, the Arduino Nano V3 combined with the expansion shield supplies power to all connected components and serves as the central data acquisition unit.

### 2.3 Analog-to-digital converter (ADC)

To overcome the Arduino Nano’s 10-bit analog-to-digital converter (ADC) resolution limit, a 16-bit ADS1115 external ADC is employed (**O**). This significantly enhances measurement precision to the sub-millivolt range. Communication between the Arduino Nano and the ADS1115 is implemented which additionally features low power consumption. The pre-existing Arduino library (ads1115_we) available online was utilized to implement the ADC module [8].

### 2.4 Light source

The development of cost-effective and portable photometers frequently relies on the use of light-emitting diodes (LEDs) as the illumination source. LEDs with narrow spectral bandwidth can eliminate the need for monochromators, thereby reducing the mechanical complexity and enhancing the device’s resistance to mechanical shock. Additional advantages of LEDs include their compact form factor, high energy efficiency, and low power consumption, which make them especially suitable for battery-powered and miniaturized systems. Consequently, multiple commercially available optical density photometers utilize LEDs as their primary light source with a spectral bandwidth comparable to that of the LED used for this photometer [2], [4].

Drawbacks of LEDs are their non-coherent emission and their broader spectral emission compared to laser diodes. These drawbacks can be partially mitigated using collimating lenses and optical bandpass filters. To obtain a light beam with a small beam angle, a 3D-printed pinhole aperture (**D**) is used (ϕ = 1.8 mm). The emission spectrum of the LED600-03 (Roithner) has its maximum near 600 nm (dominant wavelength: 597 nm, Fig. 1SI) with a full width at half maximum of 15 nm at a total radiated power of 23 mW and a beam angle of 2θ_1/2_ = 28 degrees [9]. Typical forward current of the LED600-03 is 20 mA at forward voltage of 2.1 V. In order to stabilize the LED current, it was powered via a 20 mA constant current source (KSQ2, Modellbau Schönwitz), which is connected to the Arduino Nano expansion shield. This could be replaced by a self-built constant current source.

**Fig. 2:**
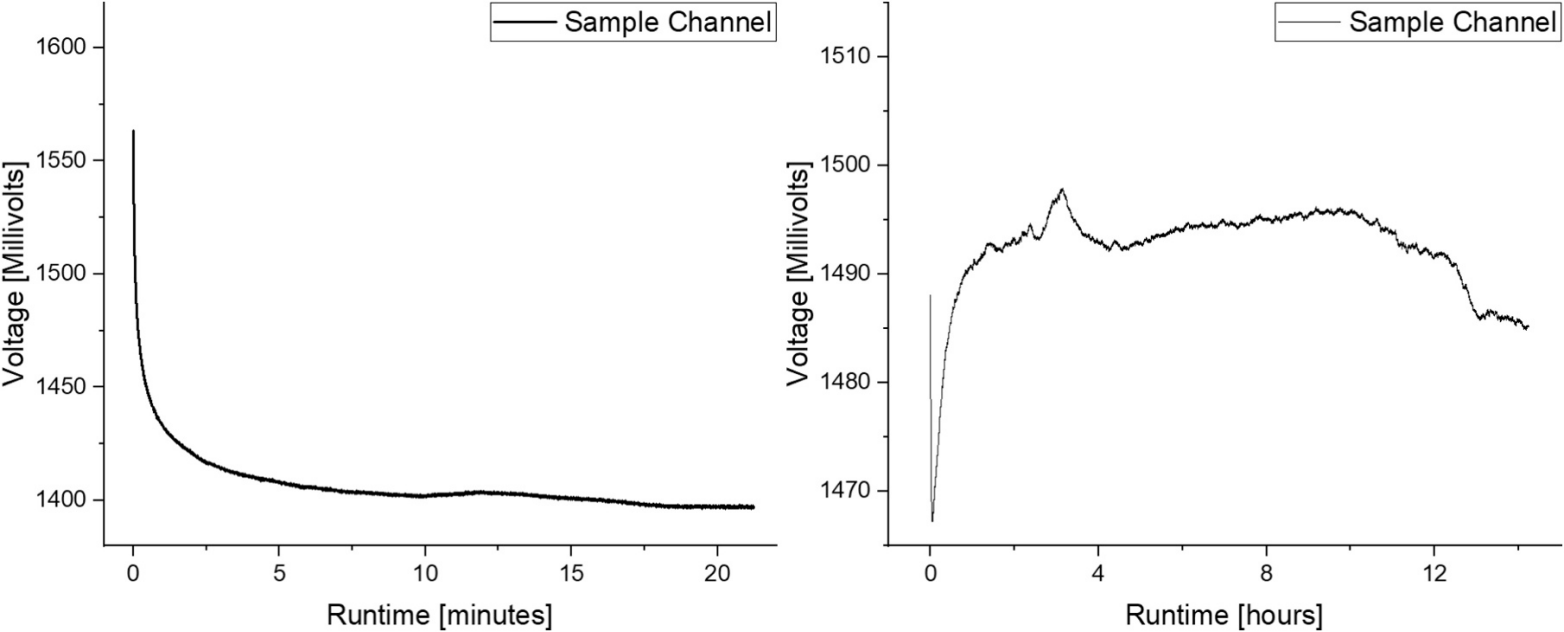
Left: Initial drift of the photometer readings after powering the photometer over 20 min via a laptop USB port. Right: Long time drift and changes of the photometer readings when powered with a power supply unit via the Arduino Nano expansion shield barrel jack over 14 h.

When powered, a rapid initial drop in light intensity within the first minutes is observed (Fig. 2, left). This is attributed to the temperature increase as the LED integrated circuit heats up, which negatively impacts the radiant intensity [9]. Additionally, photodiode voltage measurements can display variations of varying amplitude over the course of several hours (Fig. 2, right), which are primarily attributed to changes in temperature, caused by changes in the environment and secondarily by a minor degree through changes in the supply voltage. To assess the impact of these two factors, the SeCaT-meter 600 was applied under controlled temperature changes at a constant power supply (12 V / 1.0 A) inside an incubator. Increasing the set temperature of the incubator was followed by a non-linear increase in measured temperature inside the incubator, stabilizing after 100 min (Fig. 2SI). An increase in ambient temperature from 25 °C to 27 °C led to a decrease of the photodiode voltage by 20 mV per 1 °C (1308 mV at 25 °C, 1288 mV at 26 °C; 1268 mV at 27 °C).

Due to its logarithmic calculation, low optical density solutions are less affected by a 20 mV decrease of photodiode voltage due to an increase in temperature by 1 °C than high optical density solutions. Based on the previous experiment, this spans from OD_600,measured_ = 0.007 instead of OD_600, true_ = 0 up to OD_600,measured_ = 0.417 at OD_600, true_ = 0.400 (Table SI). Since bioprocesses typically reach higher optical densities, they are diluted prior to the measurement to allow for OD_600_ measurements in transmission. This would lead to an even greater error in the determined optical densities, since the faulty OD_600_ values are multiplied to account for the dilution factor of the sample.

**Fig. 3:**
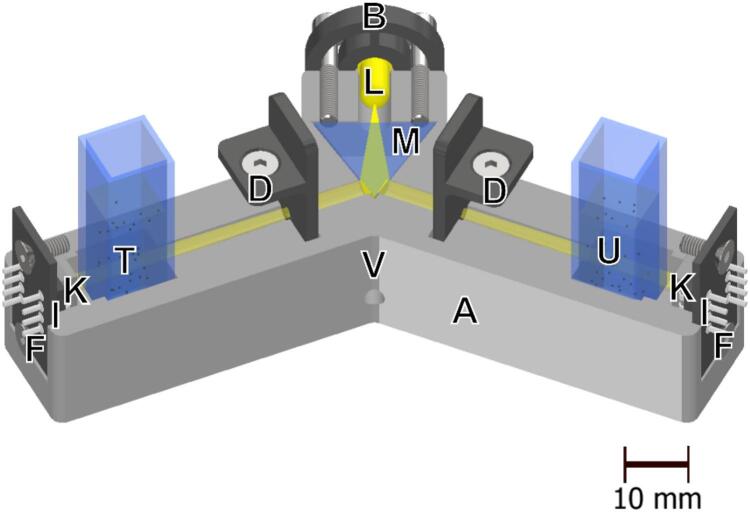
Sectional view of the main unit with an inserted cuvette, created with Autodesk inventor 2026 with annotated parts. Main unit (**A**) with cable duct (**V**), LED-mount (**B**), pinhole apertures (**D**), IC-socket holders (**F**), IC socket (**I**), OPT101 photodiode (**K**), LED600-03 (**L**), equilateral prism (**M**), cuvettes in the measurement/bottom (**T**) and reference/top channel (**U**) and cable duct in the main unit (**V**).

In a second experiment, the LED was powered at different supply voltages in the range of 12 to 7 V at a constant current of 1.0 A (Fig. 3SI) under constant temperature of 23 °C. In this range the decrease of 1 V supply voltage reduced the photodiode readings by 1 mV, which is negligible compared with the photodiode reading of 1346 mV. Voltages of 6 V and below resulted in a drastic reduction of LED intensity.

These experiments demonstrate that, in a non-temperature-controlled laboratory environment, the photometer is constantly affected by changes in the ambient temperature over the course of the day. Additionally, nearby heat sources such as pumps, refrigerators and freezers, as well as direct sunlight, influence the temperature of the device and its components. These sources of interference are especially relevant for long-running processes that are continuously monitored by OD_600_ measurements. However, since fluctuations in ambient temperature are typically neither continuously controlled nor is the turning on and off from nearby heat-sources or exposure to sunlight constantly tracked. Therefore, recalibration would need to be performed before each measurement, which would double the workload per sample. To compensate for this, a means to automatically correct these errors was investigated.

During continuous monitoring applications, such as bioprocesses, these changes can lead to inaccuracies in optical density calculations, as variations in LED intensity are superimposed on light scattering from the sample. To address this issue, the light beam is split, allowing for simultaneous reference and sample measurements to correct for light intensity changes in real time (Fig. 3). This is achieved by continuously calculating a correction factor which is the ratio from the current (*U_reference, t_*) to the initial voltage (*U_reference, 0_*) at the reference photodiode (Equation (2). This factor is applied to every measurement of the voltage at the sample photodiode (Equation (3), allowing dynamic correction of the measurement signal during operation.

(2) $$\mathrm{Correction}\;factor=\frac{U_{\mathrm{reference},\;current}}{U_{\mathrm{reference},\;\;0}}$$

(3) $$U_{\mathrm{Sample}\;Channel,\;calibrated}=\;\frac{U_{\mathrm{Sample}\;Channel,\;raw}}{\mathrm{Correction}\;factor}$$

### 2.5 Optical prism

Both the rapid initial drop and the gradual drift in LED intensity observed during extended measurements induce errors in optical density readings. To distinguish between changes in the sample's OD_600_ and fluctuations in light-source intensity, an equilateral prism (OptoSigma, DPTIH11-20-4H) was placed in the optical path at a distance of 12 mm from the LED. This optical component allows beam splitting, directing the emitted light onto two separate photodiodes (**K**), thereby allowing simultaneous detection of reference and sample signals. The prism is oriented such that one of its faces is perpendicular to the incoming LED beam (Fig. 3). According to Snell’s law, total internal reflection at the second interface, due to the high angle of incidence, causes the beam to be reflected toward the third surface of the prism, where it is refracted. This optical path yields two spatially separated light beams emerging at an angle of approximately 130°. Compared to alternative beam-splitting techniques, such as the use of optical fibers or partially reflective mirrors, equilateral prisms offer several advantages, including low loss of light intensity, mechanical robustness, geometric stability, compact form factor, and minimal maintenance. In particular, their low cost makes them attractive for integration into low-budget, portable instrumentation.

### 2.6 OPT101 photodiode

For the detection of light intensity in low-cost photometric devices, the OPT101 photodiode (Texas Instruments) represents a widely adopted solution due to its combination of performance and affordability [5], [10], [11]. This monolithic sensor integrates a large-area photodiode (5.2 mm2) with a built-in transimpedance amplifier (1 MΩ), offering a high degree of linearity between incident light intensity and output voltage [12]. The OPT101 is well-suited for portable instrumentation, as it features low quiescent current (120 µA) and low power consumption (0.6 mW). Simultaneously, it provides a high signal-to-noise ratio which is critical for accurate optical density measurements in microbial culture monitoring. Moreover, the device's compact form factor and 8-pin plastic dual in-line package (DIP) allow for easy replacement of the photodiode in the standard 8-pin IC socket.

### 2.7 SSD1306 OLED display

To allow readout from the SeCaT-meter 600 without an additional computer, the system was equipped with an SSD1306 OLED screen to display optical densities and electrical voltages from the photodiodes. The SSD1306 OLED operates efficiently on minimal current. For implementation, readily available libraries were employed, specifically *Adafruit_GFX.h* for graphical rendering [13] and *Adafruit_SSD1306.h* for device-specific control [14].

### 2.8 3D printing of photometer and the respective components

The components of the photometer, including the main unit (**A**), LED mount (**B**), prism lid (**C**), pinhole apertures (**D**), cuvette cap (**E**), IC socket holder (**F**), housing (**G**), and housing lid (**H**), were designed using *Autodesk Inventor Professional 2025*. Slicing of the models was conducted with *PrusaSlicer 2.9.1*. All components were printed via fused filament fabrication (FFF) with a *Creality K1 Max* 3D printer. 3D printing was conducted with a 0.4 mm nozzle at an extrusion temperature of 210 °C and a heat bed temperature of 55 °C. A standard printing speed of 300 mm/s, with reduced speed for external and small parts (210 mm/s) and layer height of 0.2 mm was applied. To ensure stability and precision of all components, additional settings for solid infill (10 %, 200 mm/s) and bridges (40 mm/s) were used.

For optimal performance and exclusion of ambient light, we strongly recommend the use of black polylactic acid (PLA) filament (e.g., Filament PLA Black, Verbatim, 55318, Ø = 1.75 mm). This is particularly important for printing the LED mount (**B**), pinhole apertures (**D**), and main unit (**A**), as it prevents stray light from reaching the photodiodes, thus reducing noise during readout. Pinhole apertures require black PLA to block LED radiation that may otherwise penetrate the borders of the pinhole. To account for drooping/sagging during 3D printing of the main beam paths, the optical path between the photodiode (**K**) and the prism (**M**) is corrected by drilling with a 3.5 mm drill bit. It is advised to drill the pinhole apertures (**D**) with a 1.8 mm drill bit to ensure equal diameters. All components were designed with modularity and ease of replaceability in mind, which is why they are installed and held in place with M3 screws. Mounting holes were dimensioned specifically with a 2.9 mm diameter for M3 screws, eliminating the need for threaded inserts and simplifying assembly. The sample holder is designed to be used with regular cuvettes (12 x 12 x 40 mm). The enclosure has dimensions 137 (length) x 164 (width) x 44 (height) mm and the fully assembled device weighs 300 g.

## 3 Design files summary

Design files for the dual-channel photometer (Table 2).

**Table 2:** Summary of design files used for 3d-printing of all components and system control.

Design file name	File type	Filament used [m / g]	Location of the file	License
Photometer main unit(**A**)	.stp	17 m/ 51 g	https://osf.io/beqrx	CC-By Attribution 4.0 International
LED Mount (**B**)	.stp	0.4 m / 1.3 g	https://osf.io/e7uw3	CC-By Attribution 4.0 International
Prism lid (**C**)	.stp	0.5 m / 1.6 g	https://osf.io/wx368	CC-By Attribution 4.0 International
Pinhole aperture (**D**)	.stp	1.0 m /3.0 g	https://osf.io/wa4xr	CC-By Attribution 4.0 International
Cuvette Cap (**E**), 2x	.stp	3.2 m / 10 g	https://osf.io/dkq4y	CC-By Attribution 4.0 International
IC-Socket holder (**F**)	.stp	0.5 m /1.3 g	https://osf.io/keth9	CC-By Attribution 4.0 International
Housing (**G**)	.stp	45 m / 135 g	https://osf.io/rctuh	CC-By Attribution 4.0 International
Housing lid (**H**)	.stp	11.5 m / 35 g	https://osf.io/qgd8y	CC-By Attribution 4.0 International
SeCaT-meter 600_assembly	.stp	−	https://osf.io/9a3j5	CC-By Attribution 4.0 International
Arduino_Nano_SeCaT_meter_600	.ino	−	https://osf.io/s6fzv	CC-By Attribution 4.0 International

Photometer main unit (**A**) – Main unit with sockets to insert the prism, photodiodes, LED.

LED Mount (**B**)– Holder to position the LED to the photometer housing

Prism lid (**C**) – Screw-on lid to prevent ambient light passing through the prism.

Pinhole aperture (**D**) – Insertable pinholes with 1.5 mm diameter, which are fixed in place through M3 screws.

Cuvette Cap (**E**) – Two caps to place over the cuvettes in the sample holders to prevent ambient light irradiating the photodiodes.

IC-Socket holder (**F**) – Holder for IC socket to screw into the photometer housing.

Photometer Housing (**G**) – Housing for the LED, prism, Prism lid pinhole apertures and photodiodes.

Housing lid (**H**) – Lid to close the complete photometer unit with electronic components.

SeCaT-meter 600_assembly – Model of the SeCaT-meter 600 to aid construction.

## 4 Bill of materials

Table 3

**Table 3:** Complete Bill of Materials for the SeCaT-meter 600, including all hardware components, 3D-printing filament, optical parts and electronics.

Designator	Component	Number	Cost per unit	Total cost	Source of materials	Material type
**N**	Arduino Nano V3 ATMEGA328 CH340	1	6.90 €	6.90 €	BerryBase	ElectronicsSemi-conductor
**N**	Nano Expansion Adapter – Shield for Arduino Nano	1	1.90 €	1.90 €	botland	ElectronicsSemi-conductor
**K**	OPT101P	2	8.59 €	17.18 €	Mouser	ElectronicsSemi-conductor
**F**	IC socket 8-Pin (2.54 mm pitch)	2	0.20 €	0.40 €	Reichelt	ElectronicsSemi-conductor
**O**	ADS1115 ADC Module	1	5.60 €	5.60 €	BerryBase	ElectronicsSemi-conductor
**L**	LED600-03	1	1.62 €	1.62	Roithner	ElectronicsSemi-conductor
**M**	Equilateral Prism DPTIH11-20-4H	1	70.50 €	70.50 €	OptoSigma	OpticsInorganic
**P**	OLED-Display SSD1306	1	15.39 €	15.39	Digikey	ElectronicsSemi-conductor
**R, Q**	Push button	2	3.00 €	6.00 €	Digikey	ElectronicsSemi-conductor
	Filament PLA Black Verbatim 55,318 (Ø = 1.75 mm)	238.2 g	24.95 €/kg	5.94 €	Reichelt	3D printingPolymer
	Wire (Ø 0.25 mm) various colors	1.9 *m*	0,19 €/m	0,36 €	Reichelt	ElectronicsSemi-conductor
	Shrink tube	15 cm	0,51 €/m	0,08 €	Reichelt	ElectronicsSemi-conductor
	DuPont crimp Set	1	9.75 €	9.75 €	Reichelt	ElectronicsSemi-conductor
	M3-Screws 20 mm (socket head)	2	2.14 €/ 100 pcs.	0.04 €	Schraubenluchs	Inorganic
	M3-Screws 8 mm (socket head)	1	1.67 €/ 100 pcs.	0.02 €	Schraubenluchs	Inorganic
	M3-Screws 6 mm (flat head)	11	0.69 €/ 100 pcs.	0.08 €	Schraubenluchs	Inorganic
	M2-Screws 8 mm (socket flat)	6	2.11 €/ 100pcs.	0.13 €	Schraubenbude	Inorganic
	M3-hexagonal nuts	4	0,55 €/ 100 pcs.	0.02 €	Lamprecht24	Inorganic
	M2-hexagonal nuts	6	1.00 €/ 100 pcs.	0.06 €	Schraubenhandel24	Inorganic
**S**	Constant current source 20 mA	1	1.99 €	1.99 €	Modellbau Schoenwitz	ElectronicsSemi-conductor
	Mini-B USB cable	1	1.70 €	1.70 €	Reichelt	ElectronicsSemi-conductor

We note that the system was assembled from parts that we already had at hand in our laboratory, and there is substantial scope for reducing the overall bill of materials through judicious component replacements. Cost can be drastically reduced by self-fabricating the prism either via laser cutting acrylic glass or 3D printing. However, materials of equal refractive index (n_d_ = 1.785) should be used. In total, the required components cost € 145.

## 5 Build instructions

**Fig. 4:**
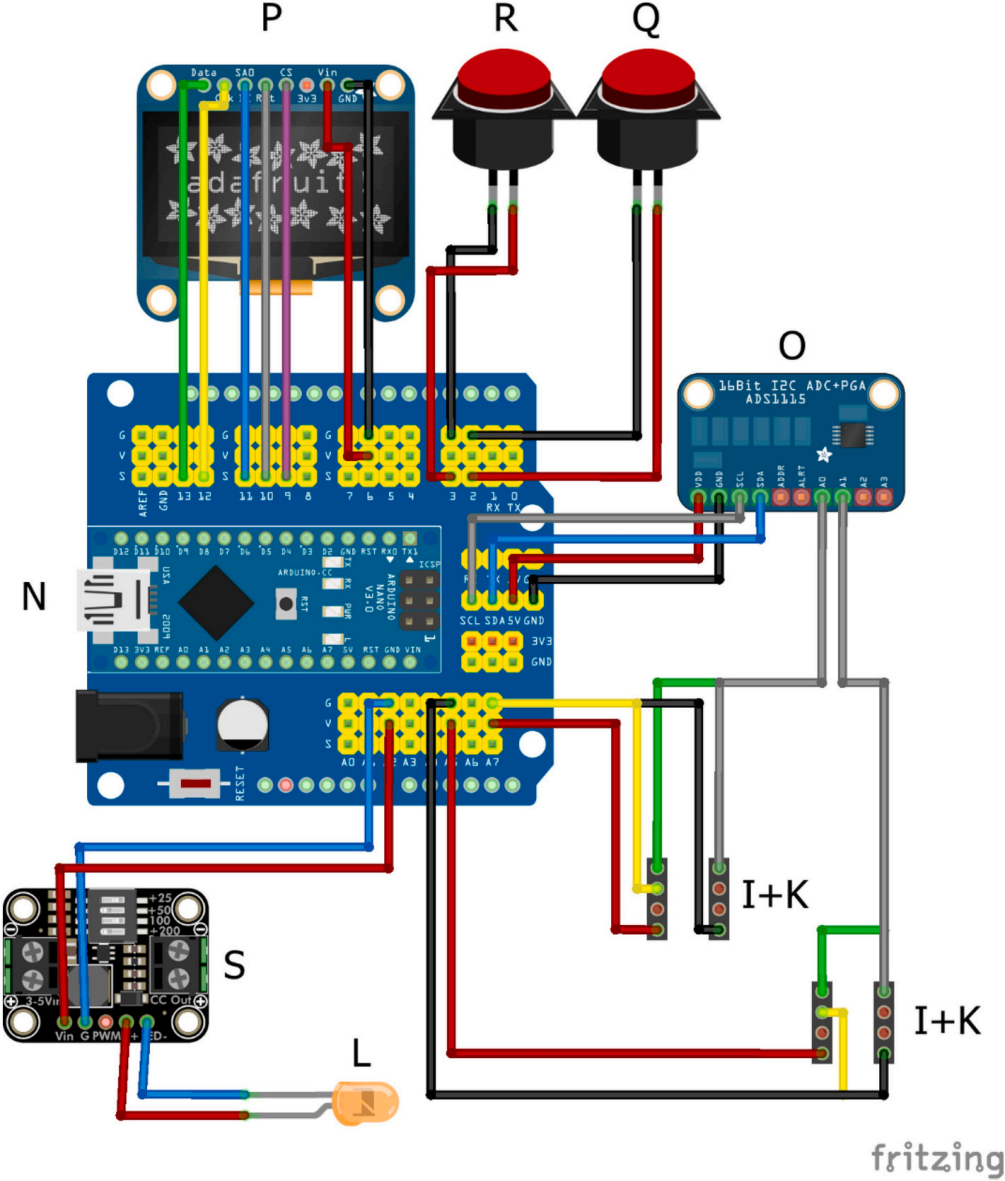
Schematic electric components with annotation and wiring of the SeCaT-meter 600, created with fritzing. IC socket (**I**), OPT101 photodiode (**K**), LED600-03 (**L**), Arduino Nano Expansion shield (**N**), ADS1115 (**O**), SSD1306 OLED (**P**), measurement/bottom channel push-button (**Q**), reference/top channel push-button (**R**), 20 mA constant current stabilization unit (**S**).

After 3D printing the main unit (**A**), LED holder (**B**), prism lid (**C**), pinhole apertures (**D**), cuvette caps (**E**), IC-socket holders (**F**), housing (**G**) and the housing lid (**H**), both pinhole apertures (**D**) are inserted and secured with M3-screws (2x 6 mm, socket flat). The 8-pin IC-sockets (**I**) are pushed into the 3D printed IC-socket holders (**F**) and wires (5 x 60 mm, 0.25 mm^2^) are soldered to the pins, as displayed in Fig. 4SI. The power supply wire (red) is crimped with a female DuPont connector and the 1 MΩ feedback (green) and output (grey) wires, as well as the common (black) and most negative power supply (yellow) wires are crimped together with female DuPont connectors. Next, the OPT101 photodiodes (**K**) are inserted into the sockets (**I**) and the assemblies are screwed into both ends of the main unit (**A**) (4x M3 x 6 mm, flat-head).

**Fig. 5:**
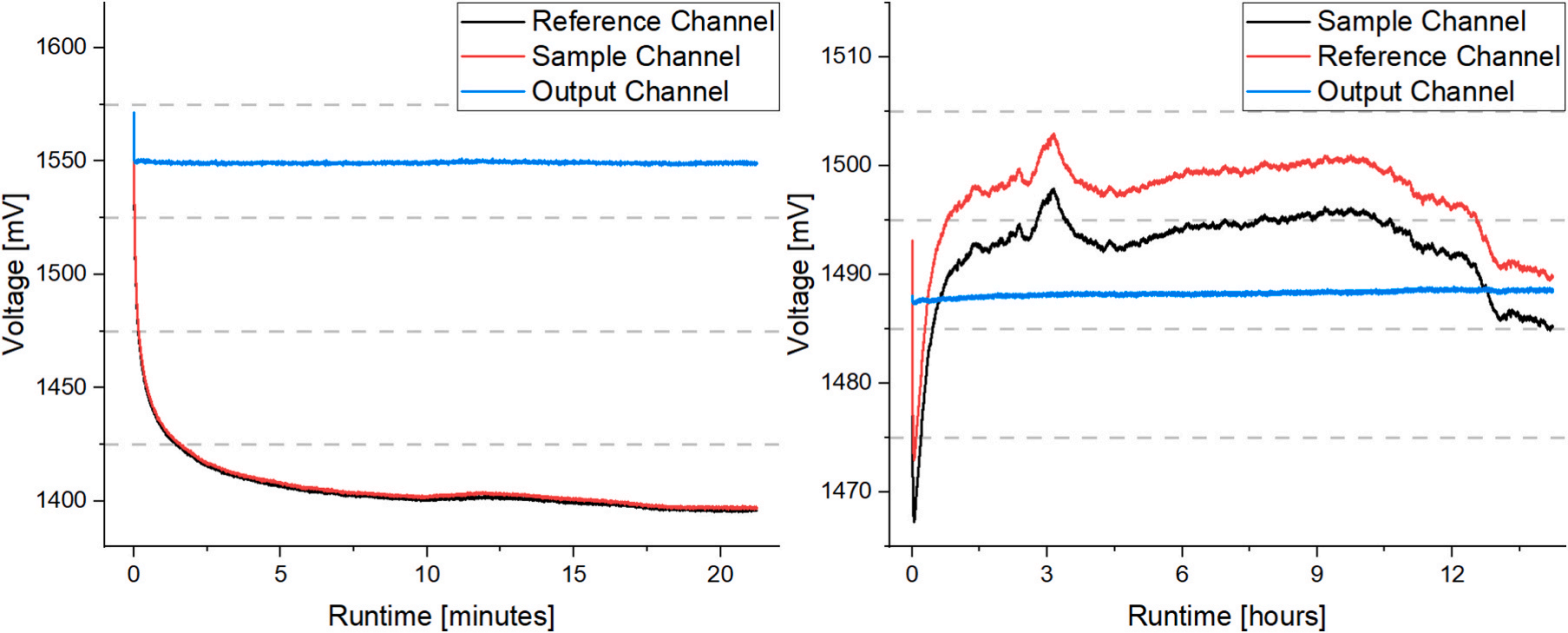
Left: Initial drift of the photometer readings after powering the photometer over 21 min, right: Long-time drift and changes of the photometer over 14 h.

The pins of the LED (**L**) are shortened and inserted into the rotatable LED holder (**B**). The LED pins are soldered wires (2x 40 mm, 0.25 mm^2^), which are then pushed through the cable duct (**V**) and soldered to the 20 mA constant current unit. Two additional wires (40 mm, 0.25 mm^2^) are soldered to V_in_ and ground on the 20 mA constant current source (**S**), crimped (female DuPont connectors) and plugged into the expansion shield. The constant current unit is then glued to the inside of the housing (**G**) to avoid loose components. Subsequently, the prism (**M**) is inserted into the triangular cavity and to avoid damaging the prism in the next step, we recommend inserting a piece of rubber, cloth or cotton into the two bottom screw holes. Next the LED with the holder is attached to the main unit and the prism is carefully locked with two screws (M3 x 20 mm, socket-head). The Arduino Nano expansion shield (**N**) is attached to the housing lid using four M3 screws (8 mm, flat-head) and four M3 hexagonal nuts. The ADS1115 ADC module (**O**) and SSD1306 OLED display (**P**) are attached to the housing lid using six M2 screws (8 mm, flat-head) and six M2 hexagonal nuts, as shown in Fig. 5SI. Additionally, the reference button (**Q**) and measurement button (**R**) are soldered with two wires each (60 mm, 0.25 mm^2^) and crimped with female DuPont connectors. Both are inserted into the lid. Wiring the ADS1115 (**O**) requires four wires (50 mm, 0.25 mm^2^) and wiring of the OLED display requires seven wires (100 mm, 0.25 mm^2^), all of which are crimped with female DuPont connectors. Lastly, wiring of the Arduino expansion shield is conducted as displayed in Fig. 4.

The radiation patterns of identical LEDs (LED600-03) exhibited large differences, which resulted in an imbalance of the electrical voltages at the photodiodes. The best autocalibration of the measurement channel diode in *Self-Calibration mode* was achieved with approximately equal electric voltages (ΔU = 10 mV) at both photodiodes. This is achieved after uploading the Arduino_Nano_SeCaT-meter_600.ino *code* to the Arduino Nano with the Arduino Integrated Development Environment (IDE) and using the *LED-Calibration mode*. Subsequently, the LED holder (**B**) is rotated until equal voltages are displayed on the OLED display (**O**). The orientation of the LED holder is then fixed with one M3-screw (10 mm, socket head). If this is not sufficient, further adjustments can be made through minor adjustments to the optical prism orientation by tightening or loosening the M3 x 20 mm, socket head screws. The assembled main unit is then pressed into the holds of the housing, and the lid is screwed to the housing. This design avoids the implementation of an additional photodiode for side-lobe measurements of LED intensity.

## 6 Operation instructions

After uploading the Arduino_Nano_SeCaT-meter_600.ino code, the photometer is ready for use once the Arduino is powered via the barrel jack or USB – either from a computer or a USB charger. The SeCaT-meter 600 possesses three different modes for calibration or measurements, *Self-Calibration Mode*, *LED Calibration Mode* and *Independent Mode*. To switch between them, the reference and measurement buttons (**R** + **Q**) need to be pressed and held simultaneously for 3 s. A successful change is indicated by the name of the new mode being displayed on screen.

**Fig. 6:**
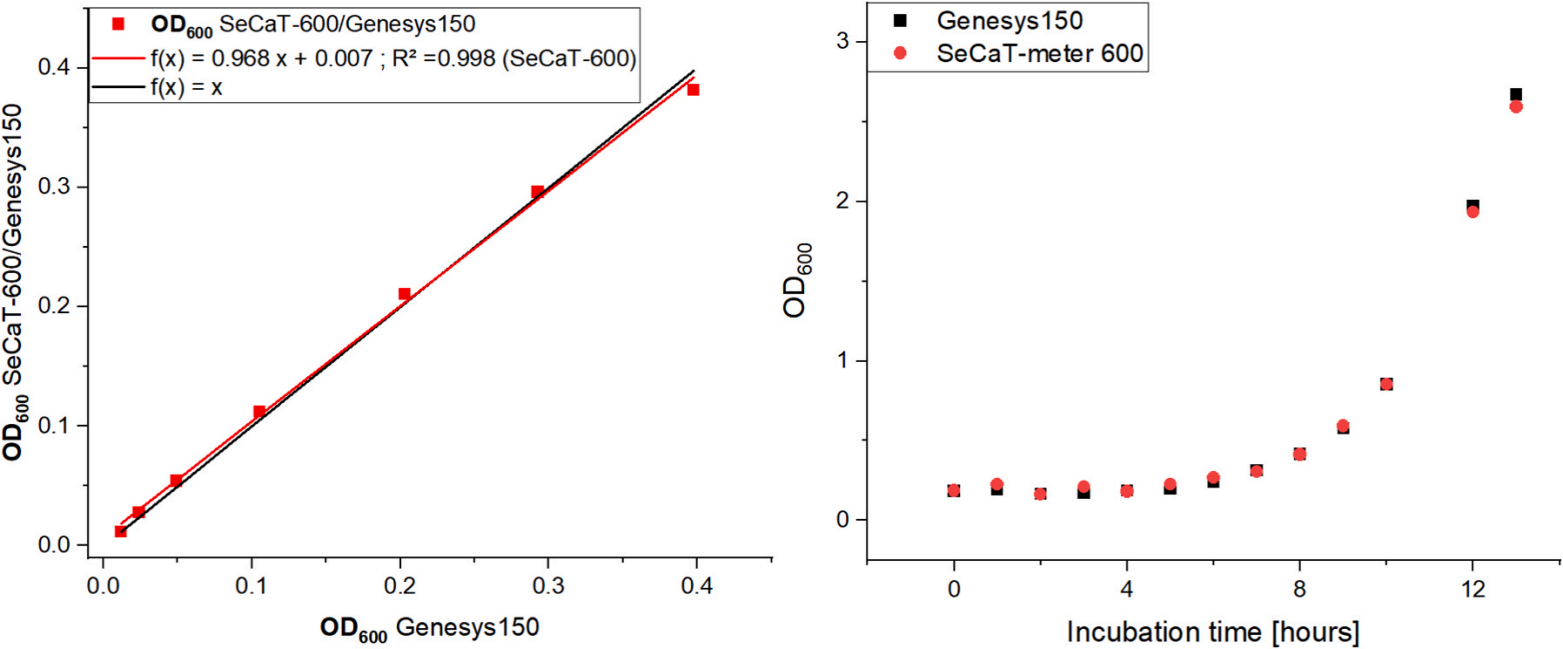
Left: Optical density at 600 nm of *Saccharomyces Cerevisiae* suspensions in water, determined with the Genesys150 (Thermo Scientific) plotted against the OD_600_ values determined with the SeCaT-meter 600 with linear regression (red). Right: OD_600_ of *S. cerevisiae* incubated at 30 °C in YPD media, measured with the Genesys150 (black) and SeCat-600 (red) within the first 13 h of incubation. (For interpretation of the references to colour in this figure legend, the reader is referred to the web version of this article.)

In *LED-Calibration Mode* electrical voltages at both photodiodes are constantly displayed on the screen to help align the LED (Fig. 6SI, center). In this mode the buttons can only be used for switching modes (press both buttons for 3 s). Both channels should be empty in this mode.

*Self-Calibration Mode* allows for immediate use of the photometer and is performed simultaneously with the sample-media calibration. For this purpose, a cuvette with the respective sample medium is inserted into the sample-channel and covered with the cuvette cap. Pressing the reference button (**R**) starts the calibration of the sample and the reference channels, which is confirmed by the message “Referencing…” on the OLED display. Upon completion, the results are displayed (Fig. 6SI, left), indicating that the sample can be removed. The button (**Q**) performs a correction of the measurement/bottom channel (**T**) readout. Pressing the reference/top channel button (**R**) performs a reference measurement of both channels which requires a reference sample solution in the bottom/measurement channel (**T**). Measurement of the bottom/measurement channel (**T**) is induced by pressing the measurement/bottom channel button (**Q**).

*Independent mode* allows to use each channel separately to measure two different samples independently from each other. A short press of the reference button (**R**) starts measurement of the top/reference channel (**U**). A long press (3 s) performs a reference measurement of the top/reference channel. To address the bottom/measurement channel (**T**), the measurement button (**Q**) needs to be pressed either long (3 s) for a reference or short for a measurement. Subsequently, the average raw data of the electrical voltage of both photodiodes, their reference values and the resulting correction factor and optical density of the measuring channel are displayed (Fig. 6SI, right). The electrical voltages at the reference and sample channels are averaged from 100 readings, performed over 2 s.

## 7 Validation and characterization

To exhibit the initial and long-term autocalibration of the sample-channel signal, the electric voltage at both photodiodes was recorded for the first 20 min of runtime and over 14 h (Fig. 5, left and right). The resulting voltages at the photodiodes of the reference and sample channels exhibited similar readings, which indicate the LED is properly aligned. To prevent the OPT101 photodiodes from producing erroneous results, each photodiode is shielded on all sides, except at the front, by the 3D-printed main unit. The front of the photodiode faces the beam channel, which is shaped as a closed tunnel to prevent infrared radiation from reaching the photodiode. This was further confirmed by inserting and removing an optical filter (Lee Filter 735 Velvet Green) in each sample compartment. The filter absorbs the LED light while allowing wavelengths above 800 nm to pass through (Fig. 7SI).

With the filter positioned in the beam path, each photodiode produced an output voltage of 7–8 mV (Fig. 8SI). This is in good agreement with the OPT101 photodiode datasheet value for the dark-error output-offset voltage (typical value: 7.5 mV) and with the values obtained when the LED was switched off. Based on these observations, near-infrared excitation of the photodiodes can be excluded. By applying the correction from equation (2), a stable output signal is generated both for the initial drift (Fig. 5, left) and for fluctuations in the photodiode signals during long measurement periods (Fig. 5, right). Early referencing during the warm-up of the device results in the greatest magnitude of error. Since OD_600_ is a logarithmic value (eq. (1) these errors drastically affect solutions with low optical density in the lag phase of microbial growth, where minute changes occur.

To determine the accuracy and precision of the SeCaT-meter 600, solutions of *Saccharomyces cerevisiae* in yeast peptone dextrose (YPD) were diluted with water. The stock solution was diluted to yield suspensions with OD_600_ values in the range of 0.01–0.40, as determined with the Genesys150 spectrophotometer (Thermo Scientific) in the OD_600_ mode. Each sample was measured 10 times (*n* = 10) with both devices after vortexing for 15 s (Fig. 6). The precision of the SeCaT-meter 600 was determined using the standard error of the mean (SEM) (Equation (4), calculated from the standard deviation *s_OD, SeCaT_* and the number of measurements *n*. Accuracy was calculated using the root mean square deviation (RMSD) (Equation (5) by comparison with the Genesys150 spectrophotometer OD_600_ results. From these calculations a RMSD of 0.008 OD_600_ and a SEM of 0.015 OD_600_ were calculated for the optical density.

(4) $$\mathrm{SEM}=\frac{s_{\mathrm{OD},SeCaT}}{\sqrt{n}}$$

(5) $$\mathrm{RMSD}=\sqrt{\frac{1}{n}*\sum_{i=1}^{n}{\left({\mathrm{OD}}_{600,SeCaT,i}-{\mathrm{OD}}_{600,Genesys,i}\right)}^{2}}$$

The linear fit of the measured data (f(x) = 0.968 x  + 0.007; R^2^ = 0.998) follows closely the identity function f(x) = x. After validation of the accuracy and precision, the SeCaT-meter 600 was used to measure the growth curve of *S. cerevisiae*. The cultivation was performed in YPD (70 ml) at 30 °C in a 250 ml Erlenmeyer flask in a shaking incubator at 220 rpm. Samples were diluted to yield suspensions with optical densities in the range of OD_600_ = 0.0–––0.4. Both the Genesys150 and the SeCaT-meter 600 were blanked against water prior to the measurement. Application of the dilution factor to the determined optical densities over 13 h of incubation time results in the typical exponential growth curve.

The natural logarithm of the recorded optical densities reveals exponential growth starting after 7 h of incubation (Fig. 9SI). Linear regression of the resulting values from seven to thirteen hours exhibits comparable slopes *m* with high precision *R^2^*, for both the commercial (*m* = 0.369 h^−1^, *R^2^* = 0.998) and the SeCaT-meter 600 photometer (*m* = 0.367 h^−1^, *R^2^* = 0.998). From these slopes a doubling time of 113 min for *S. cerevisiae* from both devices was calculated (Equation (6). Under optimal conditions, this value lies at approximately 100 min [15]. The time required for transport to the sterile bench, sampling and returning the stock solution back to the shaking incubator was circa 5 min, thus slowing the growth of *S. cerevisiae*.

(6) $$\mathrm{doubling}\;time=\frac{ln(2)}{m}$$

To conclude, the SeCaT-meter 600 allows for long time-measurements of microbial samples to determine their optical densities reliably in the range of 0.0–0.4. The prism-based approach reduces drift and sudden changes in the readout voltage at the photodiodes through usage of a reference channel. Recording the growth curve of *Saccharomyces cerevisiae* revealed a growth rate of 113 min in the exponential phase, which is in good agreement with literature reports.

## Declaration of generative AI and AI-assisted technologies in the manuscript preparation process

During the preparation of this work the authors used ChatGPT-5 in order to assist during writing the Arduino code. After using this tool/service, the authors reviewed and edited the content as needed and take full responsibility for the content of the published article.

## Ethics statements

The authors declare that this work complies with the journal ethics policies and did not involve human subjects or animal experiments.

## CRediT authorship contribution statement

**Gregor Michl:** Writing – original draft, Software, Methodology, Investigation. **Christoph Otto:** Writing – review & editing, Software. **Thomas Walther:** Writing – review & editing. **Nandor Ziebart:** Writing – review & editing, Supervision, Methodology.

## Declaration of competing interest

The authors declare that they have no known competing financial interests or personal relationships that could have appeared to influence the work reported in this paper.

## Appendix A Supplementary data

Supplementary Data 1 Experimental data and images.
