# Supplementary material for: SeCaT-meter 600: Prism-based dual channel optical density meter with self-calibration
Source: HardwareX. 2026 Aug 1;27:e00820. doi: 10.1016/j.ohx.2026.e00820 (PMC13471323; doi:10.1016/j.ohx.2026.e00820)
Supplement: Supplementary file 1 [file mmc1.docx]

**Supporting information**

Figure 1SI: Emission spectrum of LED600-03 in the range of 500 – 700 nm, recorded with HP330 Spectral Illuminance Meter, Duotone.

Figure 2SI: Photodiode readings of the SeCaT-meter 600 reference channel (black line), measurement channel (red line) and the corrected output channel (blue line) when placed inside an incubator (Infors HT Ecotron Incubator) starting from 25.3 °C over 26.3 °C to 27.3 °C. The LED and constant current supply is powered through a separate Arduino Nano Expansion shield which is powered through its barrel Jack with a laboratory compact power supply unit (Manson NSP-3630) set to 1 A and 12 V. Temperature readings inside the incubator (green dots) were performed with a Bluetooth thermometer (Govee Bluetooth Hygrometer Thermometer H5075).

Table 1SI: Calculated optical densities (OD_600,calc_) at a +1 °C and +2 °C temperature increase of the SeCaT-meter 600, reducing the photodiode voltage by 20 mV (+1 °C) and 40 mV (+2 °C) respectively to the original photodiode voltage at OD_600_ = 0.000 (1344 mV).

| OD_600,true_ (Voltage) | OD_600,calc_ +1°C (Voltage) | OD_600,calc_ +2°C (Voltage) |
| --- | --- | --- |
| 0.000 (1344 mV) | 0.007 (1324 mV) | 0.013 (1304 mV) |
| 0.100 (1068 mV) | 0.108 (1048 mV) | 0.117 (1028 mV) |
| 0.200 (848 mV) | 0.211 (828 mV) | 0.222 (808 mV) |
| 0.300 (674 mV) | 0.313 (654 mV) | 0.327 (634 mV) |
| 0.400 (535 mV) | 0.417 (515 mV) | 0.434 (495 mV) |

Figure 3SI: Photodiode readings of the SeCaT-meter 600 with the LED powered by a separate Arduino expansion shield through its barrel Jack with a laboratory compact power supply unit (Manson NSP-3630) set to an output of 1 A and 12 V (Black), 11 V (red), 10 V (blue), 9 V (green), 8 V (purple) and 7 V (yellow). At an electrical voltage of 6 V and below the LED starts to dim drastically. Experiment conducted at constant temperature of 23 °C.

Figure 4SI: Back side of the IC socket with color coded wires; power supply (red), ground (black), negative power supply (yellow), output (white), 1MΩ feedback (green).

Figure 5SI: Image of the opened SeCaT-meter 600 after assembly.

Figure 6SI: OLED-Screen outputs of *Self-Calibration Mode (left), LED Calibration Mode (center) and* *Independent mode* (right). During *Self-Calibration Mode* the electrical voltage [mV] are displayed for the measurement (Msr) of the top/reference channel (top left) and the bottom/measurement channel (bottom left) with the calculated difference D in the center. The reference values (Ref) of both channels are displayed in the center. The correction factor (CF) and the corrected optical density (OD) are displayed to the right.

Figure 7SI: Overlap of the normalized transmission, intensity and responsivity. Voltage Output of the Texas Instruments OPT101 Photodiode (black) was taken from the datasheet [12]. Normalized transmission spectrum of the Lee Filter 735 Velvet Green (green) was recorded with the Genesys150 spectrophotometer. Normalized Emission spectrum of the Roithner LED600-03 (red), recorded with HP330 Spectral Illuminance Meter, Duotone.

Figure 8SI: Electrical voltage output of each OPT101 photodiode, when a Lee Filter 735, Velvet green is inserted into the beam path of the bottom channel (black squares, 25-55 seconds) and the top channel (red triangles, 85-125 s).

Figure 9SI: Natural logarithmic values of measured optical densities at 600 nm from *Saccharomyces cerevisiae.*
